# Cluster and correspondence analyses for oral ulcer activity related factors in Behçet’s syndrome

**DOI:** 10.4317/medoral.27242

**Published:** 2025-08-16

**Authors:** Berceste Polat-Akmansoy, Burcu Aksoy, Nur Şişman-Kitapçı, Şükrü Can Akmansoy, Zehra Özge Çandereli, Fatma Büşra Sarı, Sarah Sacoor, Bindi Gokani, Azimoon Bibi, Adebowale Adesanya, Pareen Desai, Amal A Senusi, Umit Karaçaylı, Fatma Alibaz-Öner, Nevsun Inanc, Tulin Ergun, Meral Yay, Farida Fortune, Haner Direskeneli, Gonca Mumcu

**Affiliations:** 1 Assistant Professor, Department of Oral and Maxilofacial Radiology, Faculty of Dentistry, Marmara University, Istanbul, Türkiye; 2 MSc, Institute of Postgraduate Education, Istanbul University-Cerrahpasa, Istanbul, Türkiye; 3 Assistant Professor, PhD, Department of Health Management, Faculty of Health Sciences, Marmara University, Istanbul, Türkiye; 4 Assistant Professor, DDS, PhD, Department of Prosthodontics, Faculty of Dentistry, Marmara University, Istanbul, Türkiye; 5 Research Assistant, MSc, PhD, Institute of Health Sciences, Marmara University, Istanbul, Türkiye and Department of Health Management, Faculty of Economics and Administrative Sciences, Izmir Kâtip Celebi University, Izmir; 6 MSc, Institute of Health Sciences, Marmara University, Istanbul, Türkiye; 7 DDS, MDDS, Barts and The London School of Medicine and Dentistry, Centre Immunobiology and Regenerative Medicine, Queen Mary University of London, UK; 8 Barts and The London School of Medicine and Dentistry, Centre Immunobiology and Regenerative Medicine, Queen Mary University of London, UK; 9 DDS, Barts and The London School of Medicine and Dentistry, Centre Immunobiology and Regenerative Medicine, Queen Mary University of London, UK; 10 DDS, MSc, Barts and The London School of Medicine and Dentistry, Centre Immunobiology and Regenerative Medicine, Queen Mary University of London, UK; 11 DDS, PhD, Barts and The London School of Medicine and Dentistry, Centre Immunobiology and Regenerative Medicine, Queen Mary University of London, UK; 12 Professor, Department of Oral and Maxilofacial Surgery, Gülhane Faculty of Dentistry, University of Health Sciences, Ankara, Türkiye; 13 Professor, Department of Internal Medicine, Division of Rheumatology, School of Medicine, Marmara University, Istanbul, Türkiye; 14 Professor, Department of Dermatology, School of Medicine, Marmara University, Istanbul, Türkiye; 15 Associate Professor, Department of Statistics, Faculty of Science and Literature, Mimar Sinan Fine Arts University, Istanbul, Türkiye; 16 Professor, Centre Immunobiology and Regenerative Medicine, Barts and The London School of Medicine and Dentistry, Queen Mary University of London, London, UK; 17 Professor, Department of Dentomaxilofacial Radiology, Faculty of Dentistry, Istanbul Okan University, Istanbul, Türkiye

## Abstract

**Background:**

Behçet’s Syndrome (BS) is a multisystemic vasculitis characterized by a heterogeneous clinical profile, including mucocutaneous, musculoskeletal, neurological, ocular, vascular and gastrointestinal manifestations. BS patients often experience a continuous, low-level disease activity state due to persistent oral ulcers. This study aimed to define relations among oral ulcer activity (OUA), gender and treatments through K-Means Cluster and Correspondence Analyses (CA) in patients with BS.

**Material and Methods:**

In this cross-sectional study, 526 BS patients from two tertiary centres in Turkey and the United Kingdom were included. The K-Means Cluster Analysis was performed to identify homogeneous clinical profiles for OUA by combining the disease severity score reflecting organ involvement and the number of oral ulcers. Then, CA was performed to visualize associations between gender and medications (non-immunosuppressive: non-IS vs. IS) in clusters.

**Results:**

K-Means Analysis identified three clusters regarding mucocutaneous and major organ involvement and cluster regarding with major organ involvement. Clusters were named according to OUA and the disease severity.

The number of oral ulcers was found to be similar in the “Low OUA” cluster (*n*=202, 65.03%; 2.18±1.13) and the “Low OUA with Major Organ Involvement” cluster (*n*=63, 19.25%; 2.19±1.37) (*p*=0.368). These were lower than those in the “Moderate OUA” Cluster (*n*=30, 9.8%; 7.60±1.88) and the “High OUA” cluster (*n*=11, 3.59%; 14.91±2.34) (*p*<0.001).

CA visualized that non-ISs in “Low OUA” cluster, ISs in “Low OUA with Major Organ” cluster for both genders as well as male patients treated with non-ISs or ISs in “Moderate OUA” cluster were predominant groups.

**Conclusions:**

The presence of two oral ulcers might be accepted as the cut-off value for low OUA. Moreover, intensive treatment protocols must be provided for elevated oral ulcer activity in male patients who were treated with non-IS medications in BS.

** Key words:**Behçet’s syndrome, oral ulcer, k-means analysis, correspondence analysis.

## Introduction

Behçet’s Syndrome (BS) is a multisystemic vasculitis characterized by a heterogeneous clinical profile, including mucocutaneous, musculoskeletal, neurological, ocular, vascular and gastrointestinal manifestations. The remitting and relapsing nature of BS is not well understood, especially in relation to oral ulcers which are the most common clinical manifestation and a major inhibiting factor to remission in many patients (1-5). BS patients often experience a continuous, low-level disease activity state due to persistent oral ulcers (6). The integrity of the oral mucosa is compromised by the occurrence of ulcers. Oral microorganisms and their inflammatory mediators access the systemic circulation contributing to the stimulation of the immune response through this route in BS (5-7). Conventionally, patients with BS are classified into two main subgroups based on mucocutaneous symptoms and major organ involvement. Non-immunosuppressive (non-IS) medications are commonly used to control oral ulcers in mucocutaneous involvement. In cases of major organ involvement, gender and the disease course are crucial factors when treatment planning, due to the high risk of mortality and morbidity, is particularly in young male patients (5). In refractory cases of oral ulcers, ISs control disease activity and reduce inflammatory attacks as well as promoting remission in patients with major organ involvement and decrease oral ulcer activity (8). Apremilast also has a beneficial effect on decreasing of oral ulcers in BS, especially in mild disease course (9). Moreover, the number of oral ulcers may also be reduced by improving the oral health in the long term (7). Patients with poor oral hygiene may need more intensive treatments with ISs to control their oral ulcer activity as shown by the decision tree analysis (10).

Physicians customise treatment plans based on patients’ unmet needs and prognostic risk factors to prevent relapses and organ damage. As yet, "treat-to-target (T2T)" strategy for BD has not been clarified (11). Furthermore, no consensus regarding ‘oral ulcer activity’ currently exists in this perspective. Consequently, the primary study question was how to define the oral ulcer pattern within the framework of the treat-to-target strategy in this study.

K-Means Cluster Analysis, a data mining tool, uses an unsupervised learning technique to partition large datasets containing "n" data points into "k" clusters. During the clustering process, large datasets are divided into different small subgroups or clusters based on the similarity measures (12). Cluster analysis is used to define phenotypic profiles of patients to support personalized medicine and improve treatment outcomes in both paediatric BS patients (13) and adult BS patients as demonstrated in previous studies (14-17).

In cases involving complex relationships, Correspondence Analysis (CA) is used to graphically represent cross-tabulations for the analysis of categorical data (18) and is performed to identify the pattern of vascular involvement in BS (19). Therefore, this study aimed to define relations among oral ulcer activity (OUA), gender and treatment protocols through K-Means Cluster analysis and Correspondence Analysis (CA) in patients with Behçet’s Syndrome (BS).

## Material and Methods

**Figure 1:**
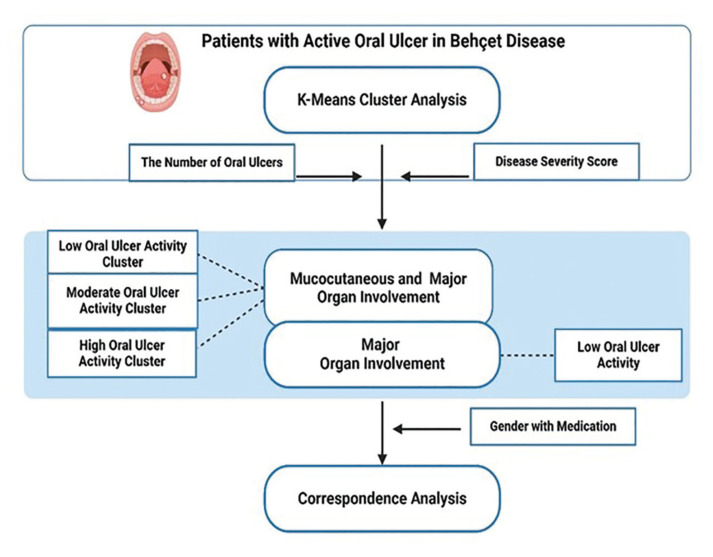
Conceptual model for the study.

In this cross-sectional study, 526 patient with Behcet’s syndrome (BS) diagnosed according to the International Study Group criteria (20) (F/M: 264/262, mean age: 37.91±10,08 years) were included from two tertiary centres in Turkey and the United Kingdom. Data were collected through clinical examinations and a questionnaire covering age, gender, organ involvement, disease duration (years), frequency of medical visits/year, treatment protocols and smoking habits (current smoker vs. non-smoker). The disease severity score reflecting organ involvement was calculated (21) and treatment protocols were classified into two groups: non-IS medications (colchicine, salazopyrin, NSAIDs, antibiotics) and IS /Immunomodulatory medications (azathioprine, corticosteroids, anti-TNF agents and interferons). The oral ulcer pattern was evaluated in order to identify homogeneous groups for oral ulcer and treatment strategies by using K-Means Cluster and Correspondence analysis (Fig. 1). The inclusion criteria required patients to be over 18 years of age and under medical control for BS.

Exclusion criteria included the presence of other chronic conditions associated with oral ulcers. The study was conducted in accordance with the principles of the Declaration of Helsinki and approved by the Ethical Committee of Marmara University Medical School (July 14, 2017; No: 09.2017.497). Informed consent was obtained from all patients.

Statistical analysis was performed using SPSS 28.0 statistic program (SPSS Inc, Chicago, IL, USA). The Chi-square test was used for categorical variables. The Kruskal Wallis test and Mann-Whitney U test were employed in non-normal distribution of continuous variables. A *p-value* of ≤0.05 was considered statistically significant.

K-Means Cluster Analysis is a machine learning method used to evaluate complex relationships among variables (12). Since organ involvement is a key point for treatment plans in BS, the analysis was performed to define patient groups with similar disease severity scores and the number of oral ulcers (Fig. 1).

In this study, these variables were first transformed into Z-scores to eliminate differences in means and variances between the variables. The analysis was then performed using the Z-scores of disease severity and the numbers of oral ulcers. The clusters were clearly visualized using a bar chart drawn by selecting the Z-scores for the severity score and number of oral ulcers variables. The elbow method and expert opinion were applied to ensure accuracy of the results. The ANOVA Table indicated that the severity score and the number of oral ulcers were significant variables for determining cluster memberships (*p*<0.001). The F-values indicated substantial differences among cluster means (*p*<0.001) as the clusters were designed to maximize these differences. K-Means Analysis identified three clusters regarding mucocutaneous and major organ involvement and one with major organ involvement (*n*=306). Clusters were named according to OUA and the disease severity. They were “Low OUA (*n*=202)”, “Low OUA with Major Organ Involvement (*n*=63) covering all patients with major organ involvement”, “High OUA (*n*=11)” and “Moderate OUA (*n*=30)” (Fig. 1, Table 1).

Correspondence Analysis (CA) aims to create interpreTable visualizations to determine complex interactions in data. The graphical presentations of CA consist of two dimensions "presenting the coordinates of points for variables (18). Gender and medication associations (male with non-IS, male with IS, female with non-IS and female with IS) in four clusters were examined using a 'Correspondence Table'. A significant association was found between rows and columns (chi-square value: 30.346, *p* <0.001). The 2D visualization captured nearly all the relationships in the dataset. In the graph, blue points (Clusters for OUA) and red points (gender with medication) were plotted.

**Figure 2:**
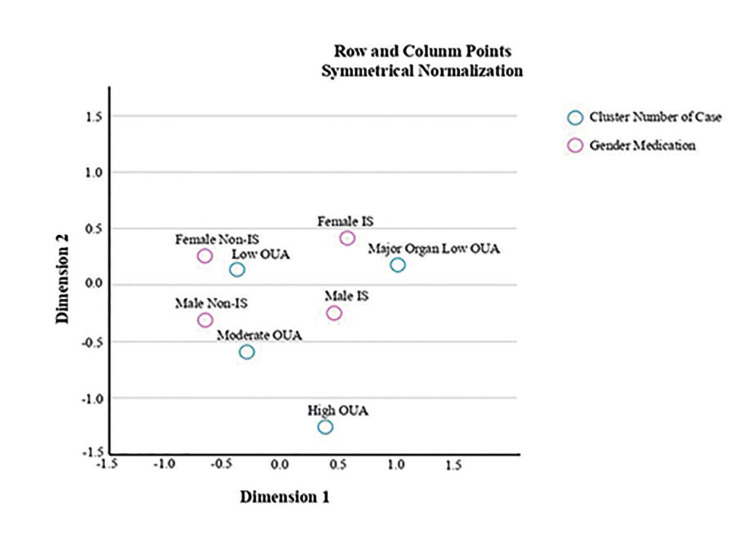
Associations between Gender with Medications and Clusters According to Correspondence Analysis.

Since the analysis visualised hidden patterns of oral ulcer, the closer points reflected strong associations whereas distant points indicated weaker associations (Fig. 1, Fig. 2).

A secondary endpoint of the study was to evaluate oral ulcer-related pain and the impact of oral ulcers on oral functions including chewing, speaking, and tasting using the Composite Index (CI) across clusters. The index is an organ specific patient-reported outcome measure, with scores ranging from “0” (inactive) to “10” during clinical examinations (22).

## Results

More than half of the group had major organ involvement (*n*=313, 59.5%) while the remaining patients had mucocutaneous and musculoskeletal involvement (*n*=213, 40.5%). The mean disease severity score reflecting organ involvement and the number of oral ulcers was found to be 5.11±1.99 and 3.15± 3.09 (*n*=306, 58.17%) in the whole group.

K-Means Analysis identified 3 clusters with mucocutaneous and major organ involvement and a cluster with major organ involvement based on the number of oral ulcers and disease severity score in BS patients (Table 1, Fig. 1). Age, gender, disease duration, the number of medical visits, healing time of oral ulcer and smoking habits of patients were found be similar in clusters (*p*>0.05) (Table 1, Table 2).

The number of oral ulcers was found to be similar in the “Low OUA” cluster (*n*=202, 65.03%; 2.18±1.13) and the “Low OUA with Major Organ Involvement” cluster (*n*=63, 19.25%; 2.19±1.37) (*p*=0.368). However, they were lower than those in the “Moderate OUA” Cluster (*n*=30, 9.8%; 7.60±1.88) and the “High OUA” cluster (*n*=11, 3.59%; 14.91±2.34) (*p*<0.001). Moreover, Composite Index scores (6.06±3.03; 6.25±2.37) were found to be similar in both low OUA clusters (*p*=0.926). Yet, these scores were significantly lower compared to those in other clusters (*p*<0.05) (Table 2). Based on these results, the presence of two oral ulcers could be considered a cut-off value to define low oral ulcer activity although the main treatment protocols in both clusters with different clinical profiles.

In the “Low OUA” cluster (F/M:106/96 mean age: 37.7±10,06 years), disease severity score was found to be 4.28±1.27. The ratios of eye and vascular involvement were found to be 19.3% and 20.8%. No significant difference was observed in the number of oral ulcers based on treatment protocols in this cluster (non-IS:2.22±1.12 vs. IS: 2.09±1.14) (*p*=0.376). Furthermore, strong associations were visualised between “Non-IS medications for both genders” (male:21.78% and female:36.14%) and the “Low OUA” cluster through the Correspondence Analysis (Table 3, Fig. 2).

The other cluster was named, “Low OUA in Major Organ Involvement” (F/M: 29/34, mean age: 38.78±9,75 years). All patients with major organ involvement (100%, Table 1) were primarily treated with IS medications in this cluster (Table 1). Patients using non-IS medications who were newly diagnosed with major organ involvement (*n*=14; F/M:9/5; 3.01±1.61) had a significantly higher number of oral ulcers compared to those treated with IS medications (*n*=49, 1.86±1.17) (*p*=0.008). In males, the number of oral ulcers was lower in IS treatment protocols (1.76±1.18) than non-IS treatment group (3.60±1.94)(p=0.012) whereas similar relation was not seen in females (*p*=0.191). As expected, the highest severity score (7.98±1.38) was observed in the “Low OUA with Major Organ Involvement” cluster compared to the other clusters (*p*<0.001) (Table 2). In Correspondence Analysis, “Treatment with ISs for both genders” (male:46.03% and female:31.75%) were visualized as noTable groups in the “Low OUA with Major Organ Involvement” cluster (Table 3, Fig. 2).

In “Moderate OUA” cluster (F/M: 12/18, mean age: 34.72±11,82 years), the number of oral ulcers and the disease severity score were 7.60±1,88 and 4.77±1,47 in the cluster. The number of oral ulcers was similar across treatments (non-IS: 7.59±1.83 vs. IS: 7.62±2.02), especially in males (*p*>0.05). Same analysis was not performed due to low number of females. A close association was visualised between the “Moderate OUA” cluster and “males treated with non-ISs and ISs” (non-ISs:30% and ISs:26.7%) in Correspondence Analysis (Table 3, Fig. 2).

The “High OUA” cluster (F/M: 8/3, mean age: 36.82±8,23 years) represented a very limited number of patients (Table 1). Although the severity score was found to be 5.27±1.73 (Table 2), the highest number of oral ulcers and Composite Index Score (14.91±2.34 and 9,45±0,68) were observed in this cluster (*p*<0.05). However, no close association was found in the Correspondence Analysis (Table 3, Fig. 2).

## Discussion

Patients with BS have heterogeneous clinical manifestations and diverse prognoses which makes it challenging to define low disease activity for treat-to-target strategies (11). In the present study, K-Means Cluster Analysis was defined as four clusters by combining oral ulcer activity and disease severity score. Then, Correspondence analysis visualised hidden patterns of oral ulcer activity and associations between gender and treatment protocols in each cluster. This is the first study in which K-Means Cluster and Correspondence analyses were used to identify oral ulcer patterns associated with the disease course, gender and treatment protocols. Gender-related differences (15), disease perception (16), the diversity of organ involvement are identified through cluster analysis to develop more effective treatment strategies in previous BS studies (14-17). Moreover, the pattern of vascular involvement was determined using the Correspondence Analysis in the previous study (19).

“Low OUA” cluster and "Low OUA with major organ involvement" cluster reflected diverse major organ involvement. The major treatment profiles were non-IS medication in the “Low OUA” cluster and ISs in the “Low OUA with major organ involvement” for both genders in the Correspondence Analysis. IS medication are used for patients with major organ involvement to decrease the mortality and morbidity. As an indirect benefit, they assist in the control of the oral ulcer activity. Besides, non-IS medication as the first-line treatment options are commonly used in mild diasese spectrum (6,23-25). As treatment plans are based on organ involvement, unmet needs and prognostic factors of patients were predicted between both clusters. Complete remission was not a realistic target for oral ulcers. According to data of these clusters, presence of two oral ulcers might be accepted as cut-off value for low oral ulcer activity. In our previous study (7), the number of oral ulcers are decreased from around 5 to 2 by performing dental and periodontal treatments and improving oral hygiene at 6-month follow-up period although systemic treatments are sTable at that period. The evidence-based data are vital for understanding patients’ needs in chronic diseases , our results offer insights for developing treatment plans and patient empowerment strategies (26) within the context of T2T strategies.

In “Moderate OUA” cluster, almost half of patients had major organ involvement. Male patients using non-IS or IS medication were visualised as prominent groups in Correspondence analysis. An increase in oral ulcer activity may indicate the potential development of major organ involvement in young males (5). Oral ulcers may result in the activation of the systemic immune response (1-5) as microorganisms, inflammatory mediators, metabolites and antigens acccess to systemic circulation through the oral ulcer where the integritiy of oral mucosa is broken. Since male patients have a higher genetic risk (27) and have more severe disease symptoms in BS (23), they may require more intensive treatments to control their oral ulcer activity within this cluster.

The “High OUA” cluster included a very limited number of patients with heterogenenous clinical profile. UnpredicTable periods of active and remission of clinical manifestations are seen in BS (3,6,8), treatment plans must be revised in relation to both oral ulcer activity and other clinical manifestations within the cluster.

The other point was that the Composite Index score as a patient-reported outcome measure was consistent with the number of oral ulcers in each cluster. The lowest scores were observed in the “Low OUA” cluster and the “Low OUA with major organ involvement” whereas the highest score was seen in “High OUA” cluster. As oral ulcers cause elevated pain and reduced oral function in BS patients (3,4), patient empowerment strategies are need to be developed to improve overall disease management (28).

This study had several strengths. It was the first study defining oral ulcer activity and disease severity score reflecting organ involvement through K-Means Cluster analysis. Secondly, Correspondence Analysis provided insight into therapeutic interventions related to gender and oral ulcers. Thirdly, the study group consisted of a large patient group from two tertiary centres in Turkey and UK. However, the cross-sectional study design and more severe patients profile in tertiary clinics were the main limitations of the study.

Consequently, the equal and less than two oral ulcers may be considered as a cut-off value for defining low oral ulcer activity in BS patients. In addition, protocols with more intensive treatment must be developed for male patients, especially those with high levels of oral ulcer activity, and were treated with non-IS medications in BS. In addition, our results may provide insights into treatment protocols and patient empowerment strategies for managing of oral ulcer activity in BS.

## Figures and Tables

**Table 1:** The Profile of Patients with BS According to Clusters Defined by K-Means Algorithm.

			Low OUA (n=202)	Low OUA in Major Organ Involvement (n=63)	High OUA (n=11)	Moderate OUA (n=30)	Oral Ulcer-Inactive (n=220)
			n (%)	n (%)	n (%)	n (%)	n (%)
Gender		Male	96 (47,5)	34 (54)	8 (72,7)	18 (60)	111 (50,5)
		Female	106 (52,5)	29 (46)	3 (27,3)	12 (40)	109 (49,5)
		Total	202 (100)	63 (100)	11 (100)	30 (100)	220 (100)
Organ Involvement		Oral ulcer	202 (100)	63 (100)	11 (100)	30 (100)	220 (100)
		Genital ulcer	170 (84,2)	48 (76,2)	8 (72,7)	25 (83,3)	170 (77,3)
		Cutaneous involvement	174 (86,1)	59 (93,7)	10 (90,9)	23 (76,7)	184 (83,6)
		Musculoskeletal inv.	92 (45,5)	40 (63,5)	6 (54,5)	16 (53,3)	104 (47,3)
		Eye involvement	39 (19,3)	55 (87,3)	5 (45,5)	13 (43,3)	87 (39,5)
		Vascular involvement	42 (20,8)	25 (39,7)	4 (36,4)	3 (10)	64 (21,9)
		Neurologic involvement	3 (1,5)	18 (28,6)	1 (9,1)	0 (0)	13 (5,9)
		Gastrointestinal inv.	11 (5,4)	12 (19)	0 (0%)	2 (6,7)	7 (3,2)
		Pathergy (+)	120 (59,4)	32 (50,8)	7 (63,6)	18 (60)	136 (61,8)
Smoking Habits		Current smoker	30 (14,85)	16 (27,1)	2 (18,2)	7 (23,33)	55 (25)
Treatment Protocols	Non-IS	Male-Non-IS	44 (37,61)	5 (35,71)	2 (50)	9 (52,94)	29 (13,2)
		Female Non-IS	73 (62,39)	9 (64,29)	2 (50)	8 (47,06)	59 (26,8)
		Total	117 (100)	14 (100)	4 (100)	17 (100)	88 (100)
	IS	Male IS	52 (61,17)	29 (59,18)	6 (85,71)	9 (69,23)	77 (35)
		Female IS	33 (38,82)	20 (40,82)	1 (14,29)	4 (30,77)	55 (25)
		Total	85 (100)	49 (100)	7 (100)	13 (100)	132 (100)

**Table 2:** Disease Related Factors According to Clusters Defined by K-Means Algorithm.

		Low OUA (n=202)	Low OUA with Major Organ Involvement (n=63)	High OUA (n=11)	Moderate OUA (n=30)	Oral Ulcer-Inactive (n=220)
		Mean±SD	Mean±SD	Mean±SD	Mean±SD	Mean±SD
Age (years)		37,7±10,06	38,78±9,75	36,82±8,23	34,72±11,82	38,32±10,03
Disease duration (years)		8,42±6,58	10,47±7,49	12,20±9,79	7,90±7,67	8,95±4,92
Number of medical visit		3,25±1,93	3,77±2,58	3,17±0,98	4,00±2,59	4,04±3,64
Severity score*		4,28±1,27	7,98±1,38	5,27±1,73	4,77±1,47	5,09±2,01
Oral Ulcer	Number **	2,17±1,13	2,11±1,35	14,91±2,34	7,60±1,88	-
	Healing time	6,78±4,44	6,84±5,35	6,64±4,71	8,47±4,70	-
PROM	Composite index***	6,25±2,37	6,06±3,03	9,45±0,68	7,80±1.21	-

*p<0.001; **p<0.001; ***p=0.014 among clusters.

**Table 3:** Assessment of Relations among Gender with Medications and Clusters According to Correspondence Analyses.

	Low OUA (n=202)	Low OUA in Major Organ Involvement (n=63)	High OUA (n=11)	Moderate OUA (n=30)
	n (%)	n (%)	n (%)	n (%)
Male with Non-IS	44 (21,78)	5 (7,93)	2 (18,18)	9 (30)
Female with Non-IS	73 (36,14)	9 (14,28)	2 (18,18)	8 (26,7)
Male with IS	52 (25,74)	29 (46,03)	6 (54,54)	9 (30)
Female with IS	33 (16,33)	20 (31,75)	1 (9,09)	4 (13,33)
Total	202 (100)	63 (100)	11 (100)	30 (100)
